# Prevalence and determinants **of** contraceptive use **among** men **in** Tanzania: Analysis **of the** 2022 demographic and health survey

**DOI:** 10.1371/journal.pgph.0005226

**Published:** 2025-09-22

**Authors:** Pankras Luoga, Jovinary Adam, Siri A. Abihudi

**Affiliations:** 1 Department of Development Studies, Muhimbili University of Health and Allied Sciences, Dar es Salaam, Tanzania; 2 An independent researcher working in Tabata, Dar es Salaam, Tanzania; 3 Institute of Traditional Medicine, Muhimbili University of Health and Allied Sciences, Dar es Salaam, Tanzania; PLOS: Public Library of Science, UNITED STATES OF AMERICA

## Abstract

Globally, contraceptive use is an important strategy in fighting maternal and neonatal deaths. The spacing and avoiding unplanned pregnancies while providing woman with enough time recovering her health and newborns growing. However, the contraception uses in developing countries including Tanzania is low and is worse among men, culturally regarded as the dominant decision makers in sexual relationships. This study intended to assess prevalence and determinants of the contraceptive use among Tanzanian men using the Tanzania Demographic and Health Survey (TDHS) 2022. The study analyzed secondary data collected using cross-sectional study design of weighted 5763 men obtained from the TDHS 2022. A dependent variable was contraceptive use and independent variables were man’s demographic and socio-economic characteristics. Bivariate and multivariable analysis were conducted and p-value<0.05 determined a significant factor. The prevalence of contraceptive use among Tanzanian men is 26%. The logistic regression showed men aged 45–49 years (aOR=3.08, 95% CI = 1.90-5.01) had higher odds to use contraceptive compared to men aged 15–19. Men with higher education (aOR=2.94, 95% CI = 1.79-4.84) had higher odds to use contraceptive compared to those with informal education, from rich quantile (aOR=1.42, 95% CI = 0.92-1.46) had higher odds compared to poor. Men with five and above children (aOR=1.62, 95% CI = 1.08-2.43) had higher odds to use contraceptive compared to those with no child. Those desired no more child had odds of 1.4 times higher to use contraceptive (aOR=1.40, 95% CI = 1.05-1.88), men who heard family planning on radio (aOR=1.39, 95% CI = 1.16-1.66) had higher odds of using contraceptive to those who did not. The contraceptive use among Tanzanian men is generally low with a prevalence rate of only 26% and was associated with man’s age, education level, wealth index, number of children, and occupation. More tailored programs targeting men to increase their education level particularly health education are crucial in increasing men’s contraceptive use in Tanzania.

## Introduction

### Background

The economic prosperity, well-being of individuals, and environmental sustainability of a nation are contingent upon its capacity to effectively manage population growth [1]. Various characteristics such as age, gender, education level, number of children a person have and wealth influence the utilization of contraceptive methods [2–5]. This has led to the promotion of diverse contraceptive methods in sub-Saharan Africa, where individuals used to have a high number of children in the past, regardless of their gender [1,6]. Recently, there has been a rise in the variety of contraceptive methods available in Tanzania, and the desire to use the methods differs across different age groups, sex and geographical location [4,7–9]. The younger population has been found to be hesitant in utilizing contraceptive methods in comparison to the elderly. There is a correlation between favorable wealth conditions and a person’s high level of education with an increased likelihood of utilizing contraception, as opposed to individuals who are impoverished and less educated [10,11]. The more the number of children a person has, the greater the likelihood that they will use a contraceptive method in both genders, in comparison to those who have none [4]. Regarding gender, research indicates that the utilization of contraception among males remains relatively low in comparison to females [3,12].

Furthermore, it is evident that having access to information on family planning has significant impact on the reproductive outcome [13]. Various individuals acquire knowledge about contraceptive from different sources such as media and hospitals [4,14,15]. However, research has revealed that men, in particular, face restricted access to certain communication channels, notably hospitals [14].

In recent times, the society has witnessed an increase in types of family planning methods in Tanzania. However, men are reported to have more knowledge and use two types of contraception, condoms and injectable [16,17]. Previous studies reported that the use of family planning without physicians’ consultations may have undesirable negative effects on the users [18]. The use and demand for FP seem to vary among ages, religion and area of residence (urban/rural) [3,18]. Tanzania Demographic and Health Survey (TDHS) reported that 38% of currently married women are using any contraceptive method, including 31% who are using any modern method and 7% of women using any traditional method. In addition, among sexually active, unmarried women, 45% use any contraceptive method, including 36% using any modern method and 8% using any traditional method [16]. However, there is limited empirical studies regarding the prevalence and factors influencing the use of contraceptive among men in the Tanzanian context. These factors can be attributed to several variables that will be revealed in the course of this study. Therefore, this study aimed to assess the prevalence and the factors associated with contraceptive uses among Tanzanian men using TDHS 2022.

## Methods and tools

This secondary study used data obtained from a cross-sectional study survey of 8^th^ Tanzania Demographic and Health Survey of 2022. In Tanzania, the program is implemented by the National Bureau of Statistics (NBS) with the financial support from the United States Agency for International Development (USAID).. The sample design for the 2022 TDHS-MIS was carried out in two stages. The first stage involved selection of sampling points (clusters) consisting of enumeration areas (EAs) delineated for the 2012 Tanzania Population and Housing Census [19]. A total of 629 clusters were selected. Among the 629 EAs, 211 were from urban areas and 418 were from rural areas. In the second stage, 26 households were selected systematically from each cluster [16].

Interviews were conducted with all women and men between the ages of 15 and 49 who were usual residents or visitors slept in the selected households the night preceding the day of the survey. No additional ethical approval was required beside the ethical considerations followed by DHS surveys. Ethical approval was obtained from participants prior to data collection by DHS program. The confidentiality and privacy of respondents are rigorously upheld throughout the DHS survey. Detailed description of the methodology and questionnaires used in the survey is available in the final report of the TDHS 2022 [16].

Concerning this study; the MEASURE DHS approved the use of the datasets after reviewing our concept note that was submitted to them. The datasets are available to the public for free at the DHS Program (https://dhsprogram.com/data/new-user-registration.cfm).

The analysis used the men’s file (MR) to elicit men’s information towards the use of contraceptive in Tanzania. In addition, this analysis involved some variables that were used in other similar studies conducted previously [8,19].

### Variables

**Fig 1:**
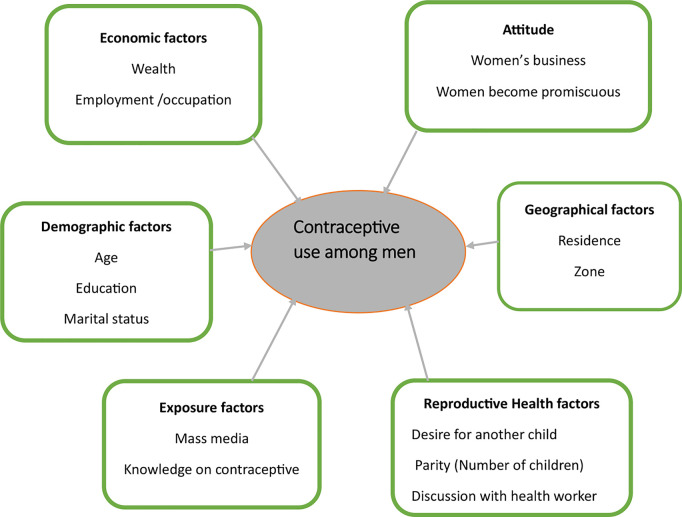
The conceptual framework of the use of contraceptive use among men.

**Independent variables** involved men’s demographic characteristics including age, education level, marital status (not in union and in union), area of residence, wealth quintile, occupation, parity, desire for another child, knowledge on contraceptive methods, heard family planning on radio, contraceptive is women’s business, women using contraception become promiscuous, watching television, discussion with health worker about contraception [8]. As indicated in (Fig 1)

**Dependent variable** was the use of the Family Planning method which was dichotomized into yes or no responses. In the analysis, all the variables with no data were treated as missing.

**Statistical analysis;** data were analyzed using Stata version 17 software with application of svy. Command to account for complex sampling design and non-response rate. This provided nationally representative estimates as per by the DHS recommendations [20]. Univariate, binary with chi-square and multivariable analysis models were used to find the association and the odds of using contraceptive based on the socio-demographic characteristics entered in the models. The chi-square was used to determine the association of the variables. Then variables which showed to be significant at p-value<0.25 in the model were tested for potential multi-collinearity to ensure the independent variables do not relate to each other except to dependent variable. Those passed the test were entered in the multivariable logistic regression analysis model to be able to control for other variables. The threshold value of p-value <0.05 was used to determine significance of the variable.

### Ethical statement

The study analyzed the collected data from Demographic Health Survey (DHS) which had already obtained ethical clearance from Tanzanian National Bureau of Statistics (NBS) for data collection hence this study did not need another ethical clearance. However, permission to use the data was requested from the DHS custodian USAID MEASURES.

## Results

### Background characteristics of study participants

The study involved 5763 male respondents, with a mean age of 29 years. Most respondents were aged 15–19 years (25.1%), followed by 20–24 years (16.2%). Majority had primary education (54.4%), with a minority having higher education (3.4%). More than a half (66.4%) residing in rural areas. More than a half (51.0%) were living in union, poorest (33.3%) richest (46.0%). More than two thirds (82.3%) had knowledge on any contraceptive method, while half (52%) listened to a radio at least once a week (Table 1**).**

**Table 1:** Socio-demographic characteristics and association with contraceptive use among men of reproductive age (15–49 years) in Tanzania (using TDHS 2022).

Variables		Frequency	Percentage	Ever used contraceptive (%)		
				No	Yes	P-value
Age	15–19	1444	25.1	90.3	9.7	<0.001
	20-24	934	16.2	69.7	30.3	
	25-29	850	14.8	69.0	31.0	
	30-34	765	13.3	67.6	32.4	
	35-39	693	12.0	68.7	31.3	
	40-44	607	10.5	71.4	28.6	
	45-49	469	8.1	68.0	32.0	
Residence	Urban	1938	33.6	71.0	29.0	0.0053
	Rural	3825	66.4	76.1	23.9	
Education	None	574	10.0	87.0	13.0	<0.001
	Primary	3134	54.4	73.7	26.2	
	Secondary	1858	32.2	73.5	26.5	
	Higher	197	3.4	55.9	44.1	
Wealth	Poor	1920	33.3	80.5	19.5	<0.001
	Middle	1191	20.7	74.6	25.4	
	Rich	2652	46.0	69.9	30.1	
Occupation	Unemployed	871	15.1	90.8	9.2	<0.001
	Employed	4892	84.9	71.5	28.5	
Marital status	Not in union	2826	49.0	77.5	22.5	**<**0.001
	In union	2937	51.0	71.4	28.6	
Parity	0	2555	44.3	81.3	18.7	<0.001
	1-2	1259	21.9	67.4	32.6	
	3-4	1038	18.0	69.7	30.3	
	5 and above	911	15.8	70.1	29.9	
Desire for another child	Have another	2213	38.4	72.8	27.2	<0.001
	Undecided	265	4.6	71.1	28.9	
	Wants no more	416	7.2	64.4	35.6	
	Sterilized	2869	49.8	77.4	22.6	
Knowledge on contraceptive methods	No	1022	17.7	90.30	9.70	<0.001
	Yes	4741	82.3	70.97	29.03	
Heard family planning on radio	No	2062	35.8	82.71	17.2	<0.001
	yes	3701	64.2	69.76	30.24	
Contraception is women’s business	Disagree	2653	46.0	73.13	26.87	<0.001
	Agree	2520	89.8	71.93	28.07	
	Don’t know	590	10.2	90.64	9.36	
Women using contraception become promiscuous	Disagree	2317	40.2	72.14	27.86	<0.001
	Agree	2881	50.0	72.47	27.53	
	Don’t know	565	9.8	93.45	6.55	
Frequency of listening radio	Not at all	1193	20.7	80.4	19.6	<0.001
	Less than once a week	1575	27.3	73.6	26.4	
	At least once a week	2994	52.0	72.4	27.6	
Frequency watching television	Not at all	1326	23.0	81.1	18.9	<0.001
	Less than once a week	1709	29.7	73.7	26.3	
	At least once a week	2728	47.3	71.6	28.4	
Discussion with health worker about contraception	No	5076	88.1	75.22	24.78	0.0011
	Yes	687	11.9	68.28	31.72	

### Prevalence of contraceptive use among men across geographical zones

**Fig 2:**
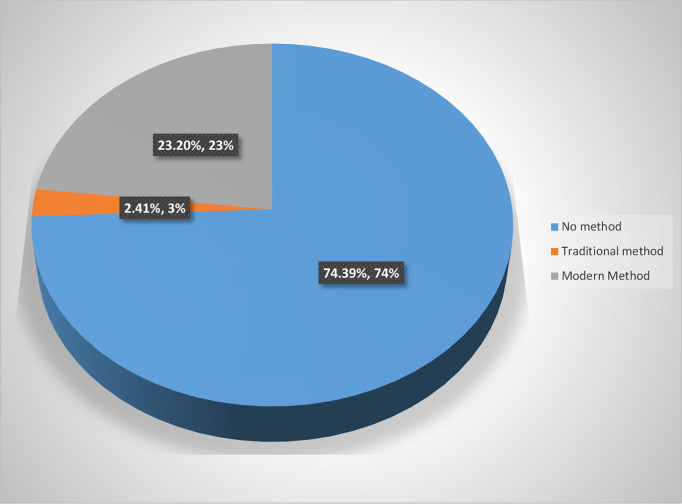
Prevalence of contraceptive use among the methods.

The overall prevalence of contraceptive use among men aged 15–49 in Tanzania is 26% (95% CI:24.02, 27.28) whereby most of them (90.6%) use modern contraceptive methods, and the rest 9.4% use traditional contraceptive methods (Fig 2**).** The prevalence varied significantly across zones. Modern contraceptive use is higher (38%) and (32%) in the Southern Highlands and South West Highlands respectively and lowest (8%) in Zanzibar. Across zones, traditional contraceptive use ranges from 0.2% to 4% in Southern and South West Highlands respectively (Table 2**).**

**Table 2:** Prevalence of the contraceptives (Modern and tradition) use among men 15-49 years across zones (Using TDHS 2022).

ZONE	NO METHOD			TRADITIONAL METHOD			MODERN METHOD		
	N	%	95% CI	N	%	95% CI	N	%	95% CI
**Overall prevalence** **26% (95% CI:24.02, 27.28)**	**4,287**	**74.4**	**72.7-75.9**	**139**	**2.4**	**1.9-2.9**	**1,337**	**23.2**	**21.7-24.8**
**Western**	419	83.6	79.78 - 86.78	9	1.7	0.72 – 4.07	74	14.7	11.58 -18.49
**Northern**	456	72.4	66.67 - 77.39	18	2.8	1.67- 4.75	157	24.8	20.00 -30.38
**Central**	417	72.2	67.77 - 76.21	15	2.6	1.34 – 5.08	145	25.2	21.46 – 29.32
**Southern Highlands**	225	59.9	54.39 - 65.18	8	2.2	1.22 – 3.77	143	37.9	32.75 – 43.43
**Southern**	222	76.3	70.18 - 81.56	1	0.2	0.20 – 1.14	68	23.5	18.32 – 29.63
**South West Highlands**	338	64.3	59.43 - 89.40	22	4.1	2.78 – 6.06	166	31.6	27.18 – 36.28
**Lake**	1,349	79.6	75.78 - 83.00	32	1.9	1.19 – 3.09	312	18.5	15.26 – 22.13
**Eastern**	691	70.8	66.91 - 74.31	28	3.0	1.74 – 4.96	257	26.3	22.91 – 30.00
**Zanzibar**	170	89.0	86.22- 91.23	6	3.0	2.04 - 4.48	15	8.0	5.88 – 10.78

### Determinants of contraceptive use among men

The results as shown in (Table 3) from logistic regression analysis revealed that the odds of that men aged 20–24 years (aOR=2.97, 95% CI = 2.19-4.04); 25–29 years (aOR=2.96, 95% CI = 2.11-4.15); 30–34 years (aOR=3.11, 2.11-4.61); 35–39 years (aOR=3.12, 95% CI = 2.07-4.71); 40–44 years (aOR=2.89, 95% CI = 1.84-4.53) and 45–49 years (aOR=3.08, 95% CI = 1.90-5.01) had higher odds to use contraceptive compared to men aged 15–19. Men with primary education (aOR=1.88, 95% CI = 1.41-2.51); secondary education (aOR=2.04, 95% CI = 1.46-2.84) and higher education (aOR=2.94, 95% CI = 1.79-4.84) had higher odds of using contraceptive compared to those with informal education. The odds of modern contraception use were significantly high among men from middle wealth index (aOR=1.23, 95% CI = 0.98-1.56) and rich (aOR=1.42, 95% CI = 0.92-1.46) compared to poor. Employed men (aOR=2.01, 95% CI = 1.34-3.02) had higher odds to use contraceptive compared to unemployed men. Compared with men with no child, men with one to two (aOR=1.51, 95% CI = 1.12-2.02) three to four (aOR=1.53, 95% CI = 1.05-2.24) and five and above (aOR=1.62, 95% CI = 1.08-2.43) children had higher odds to use contraceptives. The men who desired no more child had odds of 1.4 times higher to use contraceptive (aOR=1.40, 95% CI = 1.05-1.88), men with knowledge on contraceptive methods (aOR=1.95, 95% CI = 1.45-2.62) as well as men who heard family planning in the radio (aOR=1.39, 95% CI = 1.16-1.66). Moreover, men who didn’t perceive that a woman using contraception becomes promiscuous (aOR=0.42, 95% CI = 0.27-0.66) had lower odds to use any of the contraceptive methods compared to their counterparts.

**Table 3:** Logistic regression on demographic and socio-economic factors on the use of contraceptive method among men aged 15-49 years in Tanzania (Using TDHS 2022).

Variable		Contraceptive use among men					
		OR	95% CI	P-value	a0R	95% CI	p-value
Age	15–19	Ref			Ref		
	20-24	4.06	2.97-5.55	0.001	2.97	2.19-4.04	<0.001
	25-29	4.20	3.14-5.62	0.001	2.96	2.11-4.15	<0.001
	30-34	4.47	3.19-6.27	0.001	3.11	2.11-4.61	<0.001
	35-39	4.24	3.05-5.90	0.001	3.12	2.07-4.71	<0.001
	40-44	3.74	2.68-5.23	0.001	2.89	1.84-4.53	<0.001
	45-49	4.38	3.04-6.32	0.001	3.08	1.90-5.01	<0.001
Residence	Urban	Ref			Ref		
	Rural	0.77	0.64-0.93	0.006	0.84	0.67-1.07	0.156
Education	None	Ref			Ref		
	Primary	2.37	1.81-3.11	0.001	1.88	1.41-2.51	<0.001
	Secondary	2.40	1.79-3.23	0.001	2.04	1.46-2.84	<0.001
	Higher	5.27	3.33-8.35	0.001	2.94	1.79-4.84	<0.001
Wealth	Poor	Ref					
	Middle	1.41	1.14-1.75	0.002	1.23	0.98-1.56	0.047
	Rich	1.78	1.46-2.18	0.001	1.42	0.92-1.46	0.012
Occupation	Unemployed	Ref			Ref		
	Employed	3.92	2.61-5.89	0.001	2.01	1.34-3.02	<0.001
Marital status	Not in union	Ref			Ref		
	In union	1.38	1.17-1.63	0.001	0.60	0.27-1.32	0.21
Parity	0	Ref			Ref		
	1-2	2.10	1.78-2.49	0.001	1.51	1.12-2.02	0.006
	3-4	1.89	1.51-2.35	0.001	1.53	1.05-2.24	0.029
	5 and above	1.85	1.49-2.31	0.001	1.62	1.08-2.43	0.021
Desire for another child	Have another	Ref			Ref		
	Undecided	1.09	0.75-1.57	0.658	1.10	0.73-1.65	0.629
	Wants no more	1.48	1.14- 1.93	0.003	1.40	1.05-1.88	0.022
	Sterilized	0.78	0.66-0.932	0.006	1.08	0.58-1.88	0.650
Knowledge on contraceptive methods	No	Ref			Ref		
	Yes	3.81	2.88-5.04	0.001	1.95	1.45-2.62	<0.001
Heard family planning on radio	No	Ref					
	Yes	2.07	1.77-2.42	0.001	1.39	1.16-1.66	<0.001
Contraception is women’s business	Disagree	Ref			Ref		
	Agree	1.06	0.90-1.25	0.475	1.11	0.92-1.34	0.274
	Don’t know	0.28	0.98-0.40	0.001	0.82	0.53-1.26	0.356
Women using contraception become promiscuous	Disagree	Ref			Ref		
	Agree	0.98	0.84-1.15	0.839	0.95	0.79-1.14	0.575
	Don’t know	0.18	0.12-0.27	0.001	0.42	0.27-0.66	<0.001
Frequency of listening radio	Not at all	Ref			Ref		
	Less than once a week	1.48	1.21-1.80	0.001	0.98	0.77-1.23	0.845
	At least once a week	1.57	1.29-1.90	0.001	0.85	0.68-1.06	0.140
Frequency watching television	Not at all	Ref			Ref		
	Less than once a week	1.53	1.23	0.001	1.11	0.88-1.41	0.373
	At least once a week	1.70	1.38	0.001	1.16	0.92-1.46	0.222
Discussion with health worker about contraception	No	Ref			Ref		
	Yes	1.41	1.14-1.73	0.001	1.01	0.81-1.26	0.953

## Discussion

The study assessed the prevalence and determinants of contraceptive use among Tanzanian men. The overall prevalence of contraceptive use among men aged 15–49 in Tanzania stands at 26%, which is very low compared target of reaching 40% to Tanzania National Family Planning Costed Implementation Plan (NFPCIP) 2019–2023 [21]. Despite policy commitments under Tanzania’s NFPCIP and advocacy strategies promoting male involvement in family planning, the 26% prevalence rate of contraceptive use among men aged 15–49 remains markedly low. This figure not only lags behind the national strategic vision but also fails to meet regional and global benchmarks for equitable reproductive health engagement.

In summary, the significant factors to contraceptive use included age, education level, wealth quantile, occupation, parity and knowledge on contraceptive use. Prior descriptive statistics showed that the study population was mostly composed of young generation mostly at reproductive age as it indicated by the average age of 29 years. Majority of population found in rural area (66%), and this showed the need to focus interventions on addressing the contraceptive use challenges encountered by this group on including both those men living in union (51%) and those not living in a union (49%). The substantial proportion (80%) of the population possessed knowledge of contraception methods indicated a promising likelihood of informed decision-making and adoption of family planning methods as reported in other studies [2,17].

This emphasized the importance of education and awareness in promoting contraceptive use and responsible reproductive informed choices [14]. With high percentage (82%) of population having knowledge on contraceptive methods shows a high chance of the population making the right decision and start using family planning methods thus reducing the population [22]. Moreover, radios (52%) have demonstrated effectiveness as a platform for disseminating family planning messages in this study and other studies [4,14,23]. Hence, they may also prove beneficial in advocating for other health-related issues within communities.

Among those who use contraceptive, majority (90%) preferred modern methods including condoms in comparison with traditional methods like withdrawal. However, modern methods are reported to be more effective, reliable and increased uptake as it is supported by a study done in sub-Saharan Africa [24]. In contrast, another study found that users, particularly women, thought modern contraceptives were less efficient in preventing pregnancy and were associated with concerns regarding infertility and cancer risks [22].

Contraceptive use exhibits substantial variation among different zones in Tanzania. The Southern and South West Highlands had higher rates of modern contraception use (38% and 32%), indicating better family planning awareness and access. In contrast, Zanzibar has the lowest prevalence rate at 8%, probably due to religious views that contraceptives opposing with religious beliefs and fears of infertility [23,25]. The low level of modern contraceptive use in Zanzibar may also be influenced by limited awareness regarding the utilization of male contraceptive methods and family planning [26]In addition, the role of culture, norms and gender power dynamics may have contributed to the variation in the contraceptive use between zones [27–29].

Furthermore, it has been demonstrated that both age and educational progress influence contraceptive utilization, suggesting that maturity and educational attainment may promote family planning among Tanzanian men a pattern consistent with findings from other settings [30]. This emphasized the importance of education as a key element in improving the adoption of contraceptives and highlights the necessity for reproductive health education among adolescents to prevent unintended pregnancies [17]. The findings showed that as education increases also the use of family planning increases. This suggest that in order to improve the use of family planning among Tanzanian men increasing education is key. This finding is similar to the findings reported elsewhere [17,30]. Moreover, with advancing age, it becomes increasingly probable that a man has initiated a family and has achieved the desired number of children compared to younger individuals. Furthermore, men from moderate to affluent socioeconomic backgrounds and those who are employed demonstrate a greater inclination towards using modern contraceptives in contrast to those from less privileged backgrounds. Increased wealth and employment enhance an individual’s ability to afford modern family planning methods compared to those who are poorer or unemployed as it is supported by another study in Ghana [1]. Additionally, the majority of employed and affluent individuals are more likely to have received education, good income and exposure to family planning awareness, thereby increasing their likelihood of encountering family planning promotions [31]. This is in contrary to the study done in Ethiopia [32] where, the wealth index did not show an independent association with the use of modern contraceptives.

Moreover, having a higher number of children and expressing a desire to cease having more children correlates with increased contraceptive utilization compared to those with fewer children or no child and no such desire, a trend also observed in other studies [2,30]. This finding aligns with another study, which found that visiting family planning facilities increased the likelihood of contraceptive use among men [2]. Interestingly, men’s perception that women who use contraception are promiscuous influences their own contraceptive behavior, with those who do not hold such perceptions being less likely to utilize contraceptives. However, the study findings contradicted with the findings reported 60% contraceptive use among men in Kibaha, Tanzania [4]. Though the time of the study may have led to this remarkable difference in the prevalence as time differences among the two studies time is six years. Furthermore, the difference in coverage lie in the fact that the previous study in Kibaha focused on only one semi-urban district, whereas the current study covered the entire country, including rural, semi-urban and urban settings. Additionally, the two studies used different study populations; the Kibaha study involved only married men, whereas the current study involved the general male population across the country.

The study taps its uniqueness by assessing the use of contraceptive among men who are culturally taken to be the main decision makers and resources suppliers in the sexual relationship and family. This also accounts for all issues related to healthcare utilization, including reproductive health issues particularly decisions about whether or not to use contraceptives. This study took advantage of the gap left by many other studies that assess FP use based on women who are often reported to be merely implementers of decisions already taken by men [2,7,8,10,33].

The observed findings align with the framework used (Fig 1), highlighting the role of age, education level, wealth quantile, occupation, parity, exposure to FP via radio and knowledge on contraceptive methods in increasing contraceptive use among men. In addition, as per framework, rural residence showed to be a hindrance to contraceptive use among Tanzania men. Therefore, the alignment of findings with the framework, provides a comprehensive understanding of the factors linked to contraceptive use among men in Tanzania.

### Strengths and limitations of the study

The cross-sectional nature of the study limited the establishment of causal-effect relationships between the variables.

The study analyzed the secondary data hence could inherently take the errors that are attached to the sampling design and other biases committed during data collection. Yet, overall, the use of national survey data as well as the use of weighting offers an advantage of eliminating many biases typically associated with pooling observational data, selection, and measurement bias.

The secondary nature of the study limited the analysis only to variables that were collected during the survey. Therefore, some of the variables could not be included due to not being included in the survey though acknowledged to be linked with use of contraceptives among men.

However, the study draws its strengths in the use of large DHS dataset which enabled authors to determine national representative estimates. In addition, the use of data collected through a standardized questionnaire applied in over 90 low and middle income countries (LMICs) worldwide enhances comparability and reliability of the findings reported in the current study.

## Conclusion

The use of contraceptive among Tanzanian men is generally low 26% and its use was mostly associated with the social demographic factors of education level, type of place of residence, number of children, employment, and the wealth as well as hearing FP messages on the radio.

### Policy implications of the study findings

Based on the findings highlighted in the study, the government of Tanzania through the ministry of Health in collaboration with other stakeholders should establish tailored programs targeting men to increase their education level particularly health education focusing comprehensive reproductive health including the use contraceptive. Men should individually start to be concerned and participate in the reproductive health services like attending ANC and Postnatal care with their partners, whereby during the care, contraceptive use takes major part.

Future studies are recommended to provide detailed information on the specific types of contraceptives methods used by men in Tanzania. Additionally, qualitative studies should explore the underlying issues that prevent men from using contraceptives.
