# Supplementary material for: Prevalence and determinants of contraceptive use among men in Tanzania: Analysis of the 2022 demographic and health survey
Source: PLOS Glob Public Health. 2025 Sep 22;5(9):e0005226. doi: 10.1371/journal.pgph.0005226 (PMC12453206; doi:10.1371/journal.pgph.0005226)
Supplement: Attachment — Submitted filename: Response to Reviewers.docx [file pgph.0005226.s004.docx]

**Point by Point Response to Reviewers and Editor Comments**

Dear Sir/madam,

We are extremely thankful for the comments from the reviewers. We have addressed the concerns in a point-by-point manner and we have accordingly revised the manuscript. We highlighted the response in the response letter as well as in the manuscript.

Thank you for your constructive comments to help in improving our manuscript.

Pank

Corresponding Author.

Decision: major revision

**Reviewer 1**

1. **Summary of the research and your overall impression
   Comment:** This study is impressive and it plays a significant role of men involvement in reproductive health in a culturally sensitive societies like Tanzania where reproductive health is more termed and perceived as a women thing and not a men thing. Therefore, this study is a catalyst that portrays the essences of men involvement in reproductive health for the health and well-being of a family and a strong economic nation and if the proposed study interventions are taken into consideration by the Tanzanian government which consists of more youthful population, we are highly likely to have less childhood pregnancies' and strong families.

**Authors response: Thanks for the comment**

2. **Discussion of specific areas for improvement**
2.1Title

Comment: The Title clearly explain the study topic and scope.

**Authors response: Thank for the compliment.**

2.2 Abstract
**Comment:** The abstract is well structured with a good format flow of Background, Objectives, Methods, Results and Conclusions. However, at the background section, the authors have well elaborated the global positive implications of contraceptives with more focus on women excluding men who are crucial and of great interest for this study. Hence, the author should consider adding a brief information/explanation of the rationale of contraceptives to men for health and well-being and especially to a cultural sensitive setting like Tanzania.

**Authors response: Thank you for the comment. We have added a short description about men’s crucial role in reproductive health towards attaining reduced maternal and neonatal deaths including the use of contraceptives to promote health of women and children.**

**Comment:** The objective is clearly elaborated and the authors have tried to structure the method section by considering study type, design and data collection methods, variables and data analysis techniques. However, the authors should consider expanding briefly the data analysis techniques in line with the study objective, the author should consider a brief explanation of how prevalence was accessed then determinants of contraceptives and consider mentioning the Chi-square test and logistic regression at the abstract because there are the main and crucial test used in this study analysis to access the associations.

**Authors response: Appreciated for the comment and suggestion. We have added a short description on the Chi-square test and multivariable logistic regression analysis to enrich our method section.**

**Comment:** Therefore, the authors could consider restructuring and a little bit expanding the methods part in the abstract and here is an example of a suggestion: "This is a cross-sectional study design that deployed secondary study approach by obtaining data collected through TDHS 2022 where a total of 5763 men were obtained......... In this study, a dependent variable was contraceptive use and independent variables were men's demographic and socio-economic characteristics........ was used to examine the prevalence of contraceptive use among men while chi-square test examined the association of.... and logistic regression examined.... and p-value <0.05 determined a significant factor".

**Authors response: Appreciated for the constructive suggestion to improve our work. We considered it in our revision.**

2.3 Introduction
**Comment**: The authors should consider the following major and minor recommendations at the introduction section: Firstly, in reference to the article used to develop the idea in the first sentence “The economic prosperity, well-being of individuals, and environmental sustainability of a nation are contingent upon its capacity to effectively manage population growth”, the author should consider modifying the sentence by considering adding the trend of population growth in Africa/Sub Saharan Africa and how contraceptives can play a positive role in the economic prosperity, wellbeing of individuals and environmental sustainability.

**Authors response: Thank you for the comment and the suggestion. We ilcuded the reference and also added the information on the trends of population growth in sub-Saharan Africa as well as how contraceptives can play role in controlling the burden.**

**Comment:** For clarity reasons, the authors should consider restructuring the sentence “This has led to the promotion of diverse contraceptive methods in Sub-Saharan Africa, where individuals used to have a high number of children in the past, regardless of their gender ”, referring to the references used to develop the idea, the authors should consider restructuring this sentence by first explaining what traditional and modern contraceptive imply in Sub-Saharan Africa settings and how it has transformed over time because in the results and in the discussion part, the authors have argued about the traditional and modern contraceptives means while here in the background it is not addressed at all. For better understanding, the authors should consider re-reading the reference article no.17, a study conducted in Philippines to see how traditional and modern contraceptives are explained.

**Authors response: Thank you for the comment. We added a description on the contraceptive methods in the sub-Saharan Africa and what they imply in the setting as well as how the use has transformed over time.**

**Comment**: Besides, for the sake of clarity, the authors should consider a brief clarification of the example "where individuals used to have a high number of children in the past, regardless of their gender" by adding it up compared to today or? and if it is compared to today that people prefer few children, is it the same in rural and urban areas, given that most of our study subjects live in rural areas, and has contraception played a role in people preferring few children now compared to the past, or are there other factors that facilitate this? The authors should consider adding up a brief explanations that mention specific contraceptive methods commonly available, and mostly used in Tanzania setting before explaining the reasons for the rise in contraceptive uses in Tanzania/ before the sentence “Recently, there has been a rise in the variety of contraceptive methods available in Tanzania, and the desire to use the methods differs across different age groups, sex and geographical location ”.
For cohesion, coherence and duplication reasons, the authors should consider deleting the sentence “This study aims to investigate the factors what is behind the limited access of contraceptives for male in Tanzania”.

**Authors response: Thank you for the observation and suggestion. We deleted the sentence about the aim of the study to avoid duplication as the last sentence in the paragraph mentions about the aim of the study.**

**2.4 Methods
Comment:** Overall the statistical analysis methods chosen are appropriate to examine the research questions. However, the authors need to clarify some few major and minor issues as follows: The authors should consider adding time intervals for implementing TDHS after the sentence “In Tanzania, the program is implemented by the National Bureau of Statistics (NBS) with the financial support from the United States Agency for International Development (USAID)” because that provide a picture and backup arguments on the trend of contraceptives use over time when compared to other studies that explored the same thing (contraceptive use among men) in Tanzania using the TDHS data.
**Authors response:** **Thank you for the comment. We have specified the time for the implementation of TDHS being after every five years**.

**Comment:** The authors should consider adding explanations for the attributes/characteristics considered for the selected regions, as this may be related to why contraceptive use in Tanzania is geographically uneven.

**Authors response: Thanks for the comment and suggestion. In the TDHS all regions were included and we revised the ambiguous sentence about determining the number of regions to be involved in the survey.**

**Comment:** The authors should consider adding an explanation of what criteria/attributes/characteristics that were considered in selecting the household, as this may be related to factors to determining contraceptive use and study limitations.

**Authors response: Thank you for the comment. We added a description on the systematic selection method used to get households for interview to ensure representation.**

**Comment**: For clarity, the authors should consider adding how long the TDHS data collection took place before the paragraph “Interviews were conducted with all women and men between the ages of 15 and 49 who were usual residents or visitors slept in the selected households the night preceding the day of the survey………

**Authors response:** **Thank you for the comment and suggestion. We have added time for TDHS data collection “The study was conducted from February 14 to July 21, 2022”**

Comment: Then the authors should consider specifying what types of interviews were deployed in the TDHS, whether they were structured, unstructured or semi-structured interviews, as to some point this could determine the generalizability and validity of the data, as this information is available in the TDHS website.

**Authors response: Thank you for the comment. We added description on the use of structured interviews in the TDHS for generalizability purpose.**
**Comment:** For structure and format reasons, the authors should consider shifting this explanation “No additional ethical approval was required beside the ethical considerations followed by DHS surveys. Ethical approval was obtained from participants prior to data collection by DHS program. The confidentiality and privacy of respondents are rigorously upheld throughout the DHS survey. Concerning this study; the MEASURE DHS approved the use of the datasets after reviewing our concept note that was submitted to them” to the ethical sections.

**Authors response: Appreciated for the comment. We shifted the explanation to ethical section in the declarations.**

**Comment:** The authors have clearly explained the availability of the data used for this study by providing an accessible link and mentioning that "the datasets are available to the public free of charge from the DHS programme (<https://dhsprogram.com/data/new-user-registration.cfm>)". However, the authors did not clearly state the procedures used to obtain the data, as in the ethical issues section the authors state that "permission to use the data was requested from the DHS custodian USAID MEASURES" implying that there were procedures involved. Therefore, for reasons of reliability, the authors should consider mentioning the procedures used to acquire the data.

**Authors response: Thank you for the observation. We added a description on the procedure of submitting a concept note used to the DHS custodian in order to get permission to use the data.**
**Comment:** Based on this sentence “In addition, this analysis involved some variables that were used in other similar studies conducted previously”. For clarity reasons, the authors should further explain why they considered the variables mentioned for this study analysis, is it just because similar studies used the same variables? Why authors considered one study from Tanzania and the other from Philippines in selecting the variables for analysis?

**Authors response: Thank you for the comment. We revised a sentence and we shifted it to the description about the independent variables used in the study to give readers a more comprehensive understanding on the selection of the variables based on previous similar studies. In addition, we acknowledge the cultural differences between Tanzania and Philippines however, the factors making men use or not use contraceptives may be similar. That is why we used a study from Philippines to give a basis for selecting variables for the current study.**

**Comment**: For reasons of coherence, consistency and reliability, authors should consider moving the 'conceptual framework' to after the explanations of variables in the methods section. This should be followed by explanations of study bias. The authors should consider explaining any bias identified in this study and how the bias was addressed, because in the limitations sections the authors mentioned few biases in this study, but also, according to the STROBE checklist for cross-sectional studies, the authors should report any bias identified and explain how they addressed the bias.

**Authors response: Thank you for the comment. We deleted a sentence on bias as we found no bias in the DHS data and therefore there is no description on how they were addressed in the study.**

**Comment:** Then there is the statistical analysis section. In the statistical analysis section, and in line with the study objectives, the authors might consider first explaining what statistical methods were used to examine prevalence, as it is not mentioned before the explanations of Chi-square and logistic regressions that were used to assess the associations of numerous factors facilitating contraceptive use.

**Authors response: Thank you for the suggestion. We added description on performing descriptive analysis by calculating weighted frequencies and percentages, which allowed us to obtain the prevalence of contraceptive use among Tanzanian men and describe the background characteristics of the study participants.**

**2.5 Results**
Comment: The authors have organized the descriptive table well and the main findings comprehensively reflect the objectives of the study. However, it is unclear and not mentioned which criteria the authors of this study used to group the study subjects as poorest, middle and richest.

**Authors response: Thank you for the comment. We used re-categorised poorest and poorer to be poor, middle as middle and richest and richer to be rich. This was re-categorised based on authors’ argument that richest and richer are all rich. In addition, several DHS studies have used similar categories.**

Comment: The caption of figure 2: “Figure 2: Prevalence of contraceptive use among the methods "it is unclear with phrasing issues. Hence, should it be…. a suggestion “Prevalence of contraceptive use methods among men”.

**Authors response: Thank you for the comment and suggestion. We changed the figure 2 caption to “**Prevalence of contraceptive use methods among men” as suggested by the reviewer.

**2.6 Discussions
Comment:** The discussion comprehensively summarizes the findings of the study and links the results to other studies. However, some minor changes may be required for clarity and external validity reasons, for example, in the limitation of the study category, the sentence "The study analyzed the secondary data and therefore could inherently take the errors that are attached to the sampling design and other biases committed during data collection". The authors should consider specifically mentioning the sampling errors and biases that were committed during the data collection of this study.

**Authors response: Thank you for the comment. However, we deleted previous argument the study may have inherited some study errors or bias which were committed during the data collection of the DHS. Therefore, we avoid mentioning them as suggested by the reviewer.**

**Comment:** In addition, for improvement and external validity purposes, the authors should consider mentioning potential variables that are not included in the Tanzania Demographic Health Survey (TDHS) and other studies, but are of great importance in assessing contraceptive use among men.

**Authors response: Thank you for the comment. We added a description on the limitation of the TDHS not capturing cultural factors as they are well documented to influence men’s contraceptive uptake in various communities.**

**2.7 Conclusion
Comment:** The conclusion is consistent with the study aims and results. However, for reasons of comprehensiveness and validity, the authors should consider amending the conclusion section to include explanations of the variables that were not associated with contraceptive use among men in Tanzania.

**Authors response: Thank you for the comment. However, to our level of understanding inclusion of factors associated with contraceptive use and the recommendation on a way forward should be sufficient to make a comprehensive conclusion of the study.**

**2.8 Recommendations
Comment:** For reasons of comprehensiveness and based on the results of this study, the authors should consider proposing strategies to the government and other stakeholders to reach out to men for the health education interventions focusing on comprehensive reproductive health including contraceptive use. The strategies could be either through community outreach, as most of the study participants lived in rural areas or through media campaigns such as radio, television.

**Authors response: Thank you for the comment. We included the strategy using of using radios to promote contraceptive use among men particularly those living in rural areas.**

**2.9 Other information**
**2.9.1 Ethical issues
Comment:** Authors should consider adding explanations of how the data were handled after they were obtained from the TDHS and how long the data obtained were kept, in accordance with the Tanzanian research ethics law.

**Authors response: Thank you for the comment. However, we have not added any information on keeping the information obtained as the procedure after accessing the permission from DHS custodian to use data is to start the analysis.**

**3. Confidential comments for the editors
Comment:** The authors should consider a language expert for more language quality.

**Authors response: Thank you for the comment and we worked on it to improve the quality of language used in our work.**

**Reviewer #2: 
I. Introduction**
• Rationale:

**Comment:** The authors include all contraceptive methods (modern and traditional) without providing a clear justification. This choice is unconventional, as modern contraceptive methods are typically prioritized in public health research for their efficacy and relevance to policy. If intentional, the authors should explain why traditional methods are included. For instance, are they addressing a gap in understanding cultural preferences?! Provide a clear rationale for analyzing all contraceptive use

**Authors response: Thank you for the comment. We agree with argument made by the reviewer on the inclusion of both two types of contraceptive methods and their existing differences in terms of efficacy in protecting pregnancy. However, the main focus of the study was on the general use of contraceptives regardless of their types. Though in the study we also indicated proportion of those using modern versus traditional methods.**

• Redundancy and Lack of Focus:

**Comment:** Several points are repeated unnecessarily, such as the increase in contraceptive methods and variations by age or residence. This repetition should be streamlined. Remove redundant information and focus on factors directly related to men’s contraceptive use.

**Authors response: Thank you for the comment. We deleted the sentence only to remain with necessary information as suggested by the reviewer.**

**Comment:** The inclusion of DHS statistics on women’s contraceptive use to deviate from the main topic. The focus should be on men’s contraceptive behaviors to align with the study’s objectives.

**Authors response: Thank you for the comment. We concur with the reviewer that the use of women statistics may indicate a shift of the current study’s focus however, we did it purposively based on limited studies reporting contraceptive use among men as well as to indicate the differences in women’s statistics on the contraceptive use who are partners.**

• Language Issues:

**Comment:** Sentences like “factors what is behind the limited access of contraceptives for male” are grammatically incorrect and unclear. Phrasing should be revised for professionalism and clarity.

**Authors response: Thank you for the observation. We deleted the sentence due to repetition issue.**

**Comment:** This section lacks references to support the statements made. For example: that claim that "society has witnessed an increase in types of family planning methods in Tanzania" ; "the use of family planning without physicians’ consultations may have undesirable negative effects on the users"

**Authors response: Thank you for the comment. We referenced the statements as suggested by the reviewer.**

**II. Methods
Comment**: The methods section should clearly specify which men were included in the study (e.g., all men aged 15–49, only sexually active men) and who was asked questions about contraceptive use. It is unclear whether non-sexually active men were excluded or categorized differently, which is critical for understanding the applicability of the findings.

**Authors response: Thank you for the comment. We added in the methods section a description on the inclusion of all men aged 15-49 years as obtained from the 2022 TDHS dataset.**

**Comment:** The dependent variable is broadly defined as "use of Family Planning method,", author needs to note the difference between Family planning and contraceptive use! but also, it does not specify whether this includes modern, traditional, or all methods. Clearly define the dependent variable. If the study includes all contraceptive methods, provide a rationale for this choice and clarify how methods were categorized.

**Authors response: Thank you for the comment. We revised the definition of the dependent variable to be contraceptive use status including both traditional and modern methods. This is because the study focused on general contraceptive use. However, in the descriptive analysis we also included the prevalence for each type of contraceptive method.**

**Comment:** The study mentions that variables with missing data were treated as "missing," but it does not explain how missing data were handled in the analysis (e.g., exclusion, imputation)

**Authors response: Thank you for the comment. We added a sentence describing the exclusion of the cases with missing values on the dependent variable.**

**Comment:** The conceptual framework is presented (Figure 1), but its development and relevance to the study are not explained in detail. It is unclear how the framework was derived or why specific factors (e.g., economic, demographic, and attitudinal factors) were included. Additionally, the conceptual framework is not adequately integrated into the discussion.

**Authors response: Thank you for the comment. We added description on the development of the conceptual framework and also integrated the reflection of framework with the study findings in the discussion.**


**III. Statistical analysis
Comment**: The analysis does not mention whether it accounted for the complex survey design or applied sampling weights to ensure representativeness (e.g use of svy command). This omission is significant since DHS data typically involve stratified, clustered sampling.

**Authors response:**  Thanks for the comment. We used the “svy” command in STATA to assign the sample weight and adjust for the clustering effect and sample stratification ( page …..)
**Comment**: The study mentions collinearity testing but does not specify the cutoff value or whether any variables were excluded based on collinearity.

**Authors response:** We assessed multicollinearity among the independent variables using the variance inflation factor (VIF). None of the variables had a VIF score of 5 or higher, indicating a lack of significant collinearity. After testing for multicollinearity, there was no multicollinearity and no variables were excluded (page ….)


**IV. Results & Tables**
Comment: Table 1 is overly dense, combining socio-demographic characteristics, prevalence, and p-values in a single table.
Recommendation:
o Divide Table 1 into two separate tables:
♣ One for socio-demographic characteristics and frequencies.
♣ Another for associations with contraceptive use, including prevalence and p-values.

**Authors response: Thanks for the comment. We have divided table 1 into two separate tables, one for socio-demographic characteristics and frequencies and another for associations with contraceptive use, including prevalence and p-values.**

**Comment:** P-values and CIs in Tables 2 and 3, they are inconsistently formatted. Use a consistent format i.e. number of decimal places, across all tables

**Authors response: Thanks for the comment and suggestion. We revised the format of presenting P-values and CIs in Table 2 and 3 as suggested by the reviewer.**

**Comment:** The results do not indicate whether sampling weights or adjustments for survey design.

**Authors response:** **We used the “svy” command in STATA to assign the individual men sample weight and adjust for the clustering effect and sample stratification. This is detailed explained in the methods section specifically in the subheading “statistical analysis”**

V. Discussion
**Comment:** The discussion repeats findings unnecessarily, such as the high proportion (82%) of men with knowledge of contraceptive methods and the emphasis on education promoting contraceptive use. Streamline the discussion by consolidating similar points and avoiding repetitive statements. For instance, the impact of education could be discussed once comprehensively rather than mentioned repeatedly.

**Authors response: Thanks for the comment. We shifted the sentence on knowledge to the discussion on education and contraceptive use.**

**Comment**: Some claims lack adequate referencing (e.g., the role of radios or differences in contraceptive use over time).

**Authors response: Thanks for the comment. We included three references supporting the role of radio and mass media in contraceptive use.**

**Comment:** The discussion lacks a clear connection between the findings and actionable policy or programmatic recommendations. Translate key findings into specific recommendations, such as improving access to education, targeting rural populations, or using community leaders to address cultural attitudes.

**Authors response: Thanks for the comment. We added a description of the key findings linkage to specific recommendations of the study including increasing education particulary health education while targeting more men from rural areas.**

**Comment:** The discussion mentions contradictory findings from Kibaha, Tanzania, without exploring the reasons for these differences beyond time gaps between the studies.

**Authors response: Thanks for the comment. We added potential reasons for the differences reported in the two studies in Tanzania including coverage and study population.**

VI. Limitations
**Comment:** The discussion of biases associated with secondary data (e.g., recall bias, social desirability bias) is vague and lacks specifics on their potential impact on the findings.

**Authors response: Thanks for the comment. We deleted the sentence to avoid confusion and also to avoid repeating almost the same weakness of using secondary data.**

**Comment:** The point about the secondary nature of the data limiting the analysis is repeated unnecessarily

**Authors response: Thanks for the comment. We cleared the repetition by deleting the secondary sentence that was explaining about secondary data and bias.**


VII. Conclusion and Recommendation
**Comment:** The recommendations are vague, failing to provide specific strategies or interventions to improve contraceptive use among men.

**Authors response: Thanks for the comment. We revised the recommendation to be more focused particularly to increase education and target more men living in rural areas.**

**Comment:** Recommendation needs to be as per study findings e.g “Men should individually start to be concerned and participate in the reproductive health services like attending ANC and Postnatal care with their partners” is not part of the study findings.

**Authors response: Thank you for the comment. We revised the recommendation to focus on the study findings particularly increasing education and targeting men living in rural areas.**
VI. List of Abbreviations
**Comment**: The section is empty

**Authors response: Thank you for the observation. We included the abbreviations used in the study.**
