# Supplementary material for: Prevalence and determinants of contraceptive use among men in Tanzania: Analysis of the 2022 demographic and health survey
Source: PLOS Glob Public Health. 2025 Sep 22;5(9):e0005226. doi: 10.1371/journal.pgph.0005226 (PMC12453206; doi:10.1371/journal.pgph.0005226)
Supplement: Attachment — Submitted filename: Revised-1st draft 2024 on FP among men_20240323.docx [file pgph.0005226.s005.docx]

**Prevalence and Determinants of Contraceptive Use among Men in Tanzania: Analysis of the 2022 Demographic and Health Survey**

**PGPH-D-24-02612R1**

Dear Editor in Chief,

Thank you for considering our work including using your time to review it and ensure allocation of reviewers who provided constructive feedback to improve it. After working on the reviewers’ comments, we are now re-submitting the work for review and possibly taking a final decision. We will appreciate receiving your feedback in the near future.

Regards

Pank

| **Current** | **Suggestion** | **PI’s Response** | **Changes inserted in Page No.** |
| --- | --- | --- | --- |
| **Introduction** |  |  |  |
| **Reviewer 3** |  |  |  |
| What types of contraceptives/Family planning are preferable by men. |  | Thank you for the comment. We find the information on the types preferred by men to be important as commented by the reviewer however, we missed such information for Tanzania and got one which shows men are more knowledgeable about condoms and injectables. We added this reference. | 2 |
| **Discussion**: Show an example of those modern and traditional methods of contraceptive. |  | Thank you for the suggestion. We added examples for traditional and modern contraceptives methods. | 11 |
| **Discussion** What about culture, norms and gender that discourage men’s participating in Family planning |  | Appreciated for the comments. We added description and references on the potential reasons of culture, norms and gender power dynamics in influencing the variation of contraceptive use between geographical zones in Tanzania as suggested by the reviewer. | 11 |
|  |  |  |  |
|  |  |  |  |
| **Reviewer 4** |  |  |  |
| This study aims to investigate the factors **what is** behind the limited access of  contraceptives for male in Tanzania. | This study aims to investigate the factors behind the limited access to contraceptives for men in Tanzania. | Thank you for the comment and suggested. We deleted the sentence to avoid duplication of study objectives as suggested by the reviewer the following comment. | 2 |
| In recent times, the society has witnessed an increase in types of family planning methods in Tanzania. Previous studies reported that the use of family planning without physicians’ consultations may have undesirable negative effects on the users. The use and demand for FP seem  to vary among ages and area of residence (urban/rural). | NO reference provided | Thank you for the observation. We added references to back up the arguments as commented by the reviewer. | 2 & 3 |
| In addition, among sexually active,  unmarried women, 45% use any contraceptive method, including 36% using any modern method  and 8% using any traditional method (Tanzania Demographic and Health Survey and Malaria  Indicator Survey , 2022). | Citation style changes here, any reason? | Thank you for the observation. We changed the reference style to Vancouver style to match with the style used to the rest of the document. | 3 |
| There is this: **This study aims to investigate the factors what is behind the limited access of contraceptives for male in Tanzania.** And this: **Therefore, this study aimed to assess the prevalence and the factors associated with contraceptive**  **use among Tanzanian men using TDHS 2022.** | Is there any reason for stating the objective twice? | Appreciated for the observation. We deleted the first sentence and maintained the second as its position after the gap fits better to ensure flow of ideas. | 2&3 |
| **Methods** |  |  |  |
| Marital status | In which group did you put the widowed and divorced? I suggest you mention this. | Thank you for thoughtful question and the suggestion. We revisited the categorisations and labelled as in union and not in union including never married, widowed, divorced and separated). We are in view that the two categories are clearer than using married and not married offering contradiction of placing women who divorced and widowed. | 5, 8, 10 |
| The first stage involved the section of strata, where the number of regions required to be included in the survey were determined. The second stage involved systematic selection of households from each cluster or Enumeration Areas (EA), whereby 629 EAs were obtained. In every cluster 26 households were taken. | I suggest you use the language used in TDHS p.44. The sample design for the 2022 TDHS-MIS was carried out in two stages. The first stage involved selection of sampling points (clusters) consisting of enumeration areas (EAs) delineated for the 2012 Tanzania Population and Housing Census (2012 PHC). A total of 629 clusters were selected. Among the 629 EAs, 211 were from urban areas and 418 were from rural areas. In the second stage, 26 households were selected systematically from each cluster. | Thank you for the suggestion. We have replaced the sentence as suggested by the reviewer to easy understanding and clarity to the reader. | 3 |
| Statistical analysis | This is well described. However, I wonder why the authors did not consider multilevel modelling considering that DHS is using clusters. Reference # 15 used MLM | Thank you for the thoughtful question. We understand the concern of the reviewer on the importance of using weighting or multilevel modelling in handling complex sampling design using clusters particularly on providing national representative estimates. However, we opted for weighting using Svy. command in Stata to account for complex sampling design including clusters and non-response rate as recommended by the DHS program (<https://dhsprogram.com/Methodology/Survey-Process.cfm>) and we added a reference no 18 in the main document for the same (https://dhsprogram.com/data/Guide-to-DHS-Statistics/index.cfm). | 4 |
|  | I believe authors used this question from DHS to get the prevalence of family planning use (#418: What method did you or your partner use?). I am interested in knowing which family planning method these men used, would authors consider sharing this information in the article? The current article only talks of modern and traditional methods. | Thank you for the comment and suggestion. However, the focus of the current study was on the use of contraceptive. However, we also provided the general methods used categorised into traditional and modern methods. We understand the importance of providing the specific details on the methods used for comprehensive understanding to the reader and this gap may be left out for future studies to fill. |  |
| **Results** |  |  |  |
| had **more odds** to use contraceptive compared to men aged 15-19 | I suggest using higher odds | Appreciated for the suggestion. We replaced a word “more” with “higher” as used in formal writing as per reviewer’s suggestion. | 6 |
| Men with primary education (aOR=1.88,  95% CI=1.41-2.51); secondary education (aOR=2.04, 95% CI=1.46-2.84) and higher education (aOR=2.94, 95% CI=1.79-4.84) had odds **2.9 higher** of using contraceptive compared to those with  informal education. | Authors have provided different odds ratios, what does 2.9 odds refer to? The last group, while all others are listed. I would have said “Men with primary education (aOR=1.88,95% CI=1.41-2.51); secondary education (aOR=2.04, 95% CI=1.46-2.84) and higher education  (aOR=2.94, 95% CI=1.79-4.84) had **higher odds** of using contraceptive compared to those with informal education.” | Thanks for the comment and suggestion. We changed the sentence particularly deleted a phrase “the odds 2.9 higher” and corrected the grammar for clarity to the reader as suggested by the reviewer. | 6 |
| Table 3 | Put the overall prevalence on top of showing the zonal prevalence | Thank you for the comment and suggestion. We agree with the suggestion provided however, putting an overall prevalence of contraceptives and its three categories distorts the table and increase complex. We decided to maintain the table as all the descriptions are provided in page no. 6 under a subheading “Prevalence of contraceptive use among men across geographical zones**”** | 6 |
| **Discussion** |  |  |  |
|  | The opening statement is that the prevalence is low, but how low is it? Is there a target? The article does not share information on the expected level of FP use | Appreciated for the comment. We added a target of 40% provided in the Tanzania National Family Planning Costed Implementation Plan 2019-2023 and also gauged with international targets. |  |
|  | The opening paragraph of the discussion can be improved focusing only on the key results. The current version provided details. | Thank you for the comment and suggestion. We broke the opening paragraph of the discussion into two one about the prevalence and the second paragraph to provide summary of the key results of the study as suggested. | 10 &11 |
| Little involvement of men in family planning issues might result into over population, poor maternal and child health outcomes, and impact on family well-being (30–32). | Article #32 is on Seoul, South Korea; 2016.  32. Mosha IH, Ruben R. Communication, knowledge, social network and family planning utilization among couples in Mwanza, Tanzania- I don’t see how it would have provided information cited here. | Thank you of the observations. We deleted the whole sentence as it was providing no addition information and it was not necessary to support the study findings. | 11 |
| The substantial proportion (82%) of the  population possessed knowledge of contraception methods indicated a promising likelihood of  informed decision-making and adoption of family planning methods, potentially leading to a  reduction in population growth as reported in other studies (9,33). | Double check the citations | Thanks for the observation. Sorry for the confusion. We corrected the estimates as the reference no (2) indicate 79.9% approximately 80% of men were knowledgeable on any FP method. The reference 17 support the argument. However, we slightly rephrased the sentence to improve clarity to the reader. | 11 |
| The low level of modern contraceptive use in Zanzibar may  also be influenced by limited awareness regarding the utilization of male contraceptive methods  and family planning, as indicated by Sigalla & Charles (2013). | Improve how the citation is made | Appreciated for the comment. We changed the reference style to Vancouver for consistency. | 12 |
| Furthermore, it has been demonstrated that both age and educational progress play a role in the  utilization of contraceptives, indicating that maturity and educational attainment serve as factors  promoting family planning among Tanzanian men, as it was also reported in other studies (37). | Considering the cited paper is not in Tanzania, I suggest rephrasing: Furthermore, it has been demonstrated that both age and educational progress influence contraceptive utilization, suggesting that maturity and educational attainment promote family planning among Tanzanian men—a pattern consistent with findings from other settings (37). | Thank you for the comment and suggestion. We replaced the sentence with the suggested sentence for reader’s clarity. | 12 |
| This cater also for all issues related to health care utilization including reproductive health issues particularly on whether to use or not use contraceptives contrary to many other studies which  assess use of FP using women who are in real sense just implementers of decisions already taken by men (9,13,14,16,40). | Two things on this statement: 1. Double check the citations, 2. I don’t totally agree with the statement, particularly on are in real sense just implementers of decisions already taken by men- while I agree this is a needed study, I don’t think this is how one would show its contribution. Consider rephrasing. | Thank you for the comment. We double checked the references. We also rephrased the paragraph however, this paragraph intended to show the uniqueness of the study by taking different stand of assessing FP us among men who are culturally reported to be dominating the decisions on healthcare utilisation in the study settings. | 13 & 14 |
| The observed findings align with the framework used, highlighting the role of the role of age,  education level, wealth quantile, occupation, parity, exposure to FP via radio and knowledge on  contraceptive methods to increase the use of contraceptive among men as well as rural residence  showing to be a hindrance to contraceptive use among Tanzania men. The observed findings align  with the framework usede role of age, education level, wealth quintile, occupation, parity, and knowledge  of contraceptive use in increasing contraceptive uptake among men | Revise the paragraph | Thanks for the comment. We revised the paragraph for clarity purpose, particularly in indicating how the study findings fit in the framework indicated in figure 2. | 14 |
| **Conclusion** |  |  |  |
|  | The conclusion misses policy implications of the findings. | Thank you for the comment. We added policy implications of the study findings and deleted the sub-heading “recommendations”. | 14 & 15 |
