# Supplementary material for: Prevalence and determinants of contraceptive use among men in Tanzania: Analysis of the 2022 demographic and health survey
Source: PLOS Glob Public Health. 2025 Sep 22;5(9):e0005226. doi: 10.1371/journal.pgph.0005226 (PMC12453206; doi:10.1371/journal.pgph.0005226)
Supplement: Attachment — Submitted filename: Comments.docx [file pgph.0005226.s006.docx]

| **Current** | **Suggestion** |
| --- | --- |
| **Introduction** |  |
| This study aims to investigate the factors **what is** behind the limited access of  contraceptives for male in Tanzania. | This study aims to investigate the factors behind the limited access to contraceptives for men in Tanzania. |
| In recent times, the society has witnessed an increase in types of family planning methods in Tanzania. Previous studies reported that the use of family planning without physicians’ consultations may have undesirable negative effects on the users. The use and demand for FP seem  to vary among ages and area of residence (urban/rural). | NO reference provided |
| In addition, among sexually active,  unmarried women, 45% use any contraceptive method, including 36% using any modern method  and 8% using any traditional method (Tanzania Demographic and Health Survey and Malaria  Indicator Survey , 2022). | Citation style changes here, any reason? |
| There is this: **This study aims to investigate the factors what is behind the limited access of contraceptives for male in Tanzania.** And this: **Therefore, this study aimed to assess the prevalence and the factors associated with contraceptive**  **use among Tanzanian men using TDHS 2022.** | Is there any reason for stating the objective twice? |
| **Methods** |  |
| Marital status | In which group did you put the widowed and divorced? I suggest you mention this. |
| The first stage involved the section of strata, where the number of regions required to be included in the survey were determined. The second stage involved systematic selection of households from each cluster or Enumeration Areas (EA), whereby 629 EAs were obtained. In every cluster 26 households were taken. | I suggest you use the language used in TDHS p.44. The sample design for the 2022 TDHS-MIS was carried out in two stages. The first stage involved selection of sampling points (clusters) consisting of enumeration areas (EAs) delineated for the 2012 Tanzania Population and Housing Census (2012 PHC). A total of 629 clusters were selected. Among the 629 EAs, 211 were from urban areas and 418 were from rural areas. In the second stage, 26 households were selected systematically from each cluster. |
| Statistical analysis | This is well described. However, I wonder why the authors did not consider multilevel modelling considering that DHS is using clusters. Reference # 15 used MLM |
|  | I believe authors used this question from DHS to get the prevalence of family planning use (#418: What method did you or your partner use?). I am interested in knowing which family planning method these men used, would authors consider sharing this information in the article? The current article only talks of modern and traditional methods. |
| **Results** |  |
| had **more odds** to use contraceptive compared to men aged 15-19 | I suggest using higher odds |
| Men with primary education (aOR=1.88,  95% CI=1.41-2.51); secondary education (aOR=2.04, 95% CI=1.46-2.84) and higher education (aOR=2.94, 95% CI=1.79-4.84) had odds **2.9 higher** of using contraceptive compared to those with  informal education. | Authors have provided different odds ratios, what does 2.9 odds refer to? The last group, while all others are listed. I would have said “Men with primary education (aOR=1.88,95% CI=1.41-2.51); secondary education (aOR=2.04, 95% CI=1.46-2.84) and higher education  (aOR=2.94, 95% CI=1.79-4.84) had **higher odds** of using contraceptive compared to those with informal education.” |
| Table 3 | Put the overall prevalence on top of showing the zonal prevalence |
| **Discussion** |  |
|  | The opening statement is that the prevalence is low, but how low is it? Is there a target? The article does not share information on the expected level of FP use |
|  | The opening paragraph of the discussion can be improved focusing only on the key results. The current version provided details. |
| Little involvement of men in family planning issues might result into over population, poor maternal and child health outcomes, and impact on family well-being (30–32). | Article #32 is on Seoul, South Korea; 2016.  32. Mosha IH, Ruben R. Communication, knowledge, social network and family planning utilization among couples in Mwanza, Tanzania- I don’t see how it would have provided information cited here. |
| The substantial proportion (82%) of the  population possessed knowledge of contraception methods indicated a promising likelihood of  informed decision-making and adoption of family planning methods, potentially leading to a  reduction in population growth as reported in other studies (9,33). | Double check the citations |
| The low level of modern contraceptive use in Zanzibar may  also be influenced by limited awareness regarding the utilization of male contraceptive methods  and family planning, as indicated by Sigalla & Charles (2013). | Improve how the citation is made |
| Furthermore, it has been demonstrated that both age and educational progress play a role in the  utilization of contraceptives, indicating that maturity and educational attainment serve as factors  promoting family planning among Tanzanian men, as it was also reported in other studies (37). | Considering the cited paper is not in Tanzania, I suggest rephrasing: Furthermore, it has been demonstrated that both age and educational progress influence contraceptive utilization, suggesting that maturity and educational attainment promote family planning among Tanzanian men—a pattern consistent with findings from other settings (37). |
| This cater also for all issues related to health care utilization including reproductive health issues particularly on whether to use or not use contraceptives contrary to many other studies which  assess use of FP using women who are in real sense just implementers of decisions already taken by men (9,13,14,16,40). | Two things on this statement: 1. Double check the citations, 2. I don’t totally agree with the statement, particularly on are in real sense just implementers of decisions already taken by men- while I agree this is a needed study, I don’t think this is how one would show its contribution. Consider rephrasing. |
| The observed findings align with the framework used, highlighting the role of the role of age,  education level, wealth quantile, occupation, parity, exposure to FP via radio and knowledge on  contraceptive methods to increase the use of contraceptive among men as well as rural residence  showing to be a hindrance to contraceptive use among Tanzania men. The observed findings align  with the framework usede role of age, education level, wealth quintile, occupation, parity, and knowledge  of contraceptive use in increasing contraceptive uptake among men | Revise the paragraph |
| **Conclusion** |  |
|  | The conclusion misses policy implications of the findings. |
